# Construction of Interferon-Gamma-Related Gene Signature to Characterize the Immune-Inflamed Phenotype of Glioblastoma and Predict Prognosis, Efficacy of Immunotherapy and Radiotherapy

**DOI:** 10.3389/fimmu.2021.729359

**Published:** 2021-09-10

**Authors:** Hang Ji, Yixu Ba, Shuai Ma, Kuiyuan Hou, Shan Mi, Xin Gao, Jiaqi Jin, Qin Gong, Ting Liu, Fang Wang, Zhihui Liu, Shupeng Li, Jianyang Du, Shaoshan Hu

**Affiliations:** ^1^Department of Neurosurgery, The Second Affiliated Hospital of Harbin Medical University, Harbin, China; ^2^Translational Medicine Research and Cooperation Center of Northern China, Heilongjiang Academy of Medical Sciences, Harbin, China; ^3^The Key Laboratory of Myocardial Ischemia, Ministry of Education, Harbin, China; ^4^School of Life Sciences, Nanjing University, Nanjing, China; ^5^Faculty of Pharmacy, Harbin Medical University (DAQING), Daqing, China; ^6^Department of Neurosurgery, Shandong Provincial Hospital Affiliated to Shandong First Medical University, Jinan, China; ^7^Department of Neurosurgery, Emergency Medicine Center, Zhejiang Provincial People’s Hospital Affiliated to Hangzhou Medical College, Hangzhou, China

**Keywords:** glioblastoma, interferon-gamma, tumor immune microenvironment, IFNG-related gene signature, anti-tumor immune response, immune checkpoint blockade therapy, radiotherapy

## Abstract

Interferon-gamma (IFNG) has profound impacts on tumor-immune interaction and is of great clinical significance for multiple cancers. Exploring the role of IFNG in glioblastoma (GBM) may optimize the current treatment paradigm of this disease. Here, multi-dimensional data of 429 GBM samples were collected. Various bioinformatics algorithms were employed to establish a gene signature that characterizes immunological features, genomic alterations, and clinical characteristics associated with the IFNG response. In this way, a novel IFNG-related gene signature (IFNGrGS, including TGFBI, IL4I1, ACP5, and LUM) has been constructed and validated. Samples with increased IFNGrGS scores were characterized by increased neutrophil and macrophage infiltration and exuberant innate immune responses, while the activated adaptive immune response may be frustrated by multiple immunosuppressive mechanisms. Notably, the IFNG pathway as well as its antagonistic pathways including IL4, IL10, TGF-beta, and VEGF converged on the expression of immune checkpoints. Besides, gene mutations involved in the microenvironment were associated with the IFNGrGS-based stratification, where the heterogeneous prognostic significance of EGFR mutation may be related to the different degrees of IFNG response. Moreover, the IFNGrGS score had solid prognostic value and the potential to screen ICB and radiotherapy sensitive populations. Collectively, our study provided insights into the role of IFNG on the GBM immune microenvironment and offered feasible information for optimizing the treatment of GBM.

## Introduction

Glioblastoma (GBM) is the most frequent primary brain malignancy. Current treatments, including surgery, radiotherapy, chemotherapy, targeted therapy, and tumor treatment field, remain palliative for most patients (1, 2). The median survival of GBM sufferers is 14.4 months, with only 9% for their 5-year survival rate (3). It is an urgent and challenging issue to improve the current treatment of GBM.

Nowadays, advances in the field of tumor immunology have culminated in several promising immunotherapies. For instance, immune checkpoint blockade (ICB) therapy has greatly prolonged the overall survival of patients suffering from melanoma, lung cancer, breast cancer, and other tumors by relieving the redundant inhibition of the anti-tumor immune response (4–6). Also, the chimeric antigen receptor (CAR) T-cell therapy has achieved durable tumor control in various cancers by enhancing the anti-tumor immune activity (7, 8). However, immunotherapies have encountered waterloo in the treatment of GBM due to the blood-brain barrier, the special cellular composition, and the inefficient immune ‘afferent’ and ‘efferent’ structures in the central nervous system (CNS) (9–12). In addition, the current preclinical models have limitations in mimicking the dynamics of tumor-immune interaction during tumor evolve (13, 14). Therefore, there remains a need to comprehensively understand the role of the immune system in the specific microenvironment of GBM.

IFNG plays a vital role in orchestrating both innate and adaptive immune responses (15, 16). Accumulative evidence has suggested that IFNG activates the anti-tumor immune response through enhancing antigen presentation, T-lymphocyte differentiation and maturation, killing of tumor cells, and suppressing regulatory T cells in various cancers (16–18). In addition to reflecting the spontaneous immune-activated status of the tumor, gene signatures related to IFNG response are widely associated with the expression of immune checkpoints, tumor mutational burden (TMB), responsiveness to immunotherapy, and clinical outcomes of various cancers (19, 20). Although studies have confirmed the cytotoxicity of IFNG on GBM cells (21, 22), the immunomodulatory effect of IFNG in the GBM microenvironment has been less elucidated. Besides, a clinical trial has reported that IFNG maintenance therapy failed to benefit GBM patients (23), indicating that our understanding of the role of IFNG in GBM, as well as its clinical significance, remains inadequate. Recently, high-dimensional data have advanced human understanding of tumor-immune interaction to an unprecedented depth at the pan-cancer scale, such as the proposition of the 6 immune subtypes of tumors and correlating T cell function with various genomic, transcriptomic, and epigenetic features (24–26). Here, we build on these efforts to decode the role of the IFNG response in the specific immune microenvironment of GBM and the chain reactions it triggers.

To comprehensively elucidate the IFNG-related immunological features in GBM, we constructed a novel IFNG-related gene signature (IFNGrGS) for characterizing the IFNG response in GBM and compared it with the previously pan-cancer-based IFNG gene signatures. Through correlating the IFNGrGS with tremendous innate and adaptive immunological molecules, cells, signaling pathways, biological processes, and tumor genomic alterations, we revealed that increased IFNG responses may indicate an immune-inflamed microenvironment, in which innate immune responses were exuberant while activated adaptive immune responses were inhibited by a variety of immunosuppressive mechanisms. Notably, both the IFNG response and the signaling pathways that antagonize IFNG may be involved in the expression of multiple immune checkpoints. In addition, our constructed IFNGrGS-based stratification excelled in predicting prognosis, ICB responsiveness, and radiotherapy efficacy, providing a viable reference for optimizing GBM treatment.

## Materials and Methods

### Data Collection and Pre-processing

The mRNA sequencing data of a total of 429 GBM samples and corresponding demographics were involved in this study, of which 166 were retrieved from The Cancer Genome Atlas (TCGA) database (https://portal.gdc.cancer.gov/) and the rest from the Chinese Glioma Genome Atlas (CGGA) database (139 from the mRNA-seq CGGA325 cohort and 124 from the microarray CGGA301 cohort) (http://www.cgga.org.cn/). Data sets were cleaned and normalized separately. The mRNA-seq data was TPM normalized and microarray data was log-transformed. The somatic mutation and copy number variation profiles of GBM were retrieved from the TCGA database. Gene sets involved in this study were retrieved from the Molecular Signatures Database (MSigDB, http://www.gsea-msigdb.org/gsea/msigdb, v7.3) and previous studies, and were organized as **Supplementary File 1** (19, 27, 28).

### Gene Set Enrichment Analysis (GSEA) and Single-Sample Gene Set Enrichment Analysis (ssGSEA)

GSEA analysis was conducted using the software GSEA (v4.0.3) based on gene sets derived from MSigDB database (v7.3). Besides, the activation of signaling pathways of interest was assessed by calculating their ssGSEA score based on the R package ‘GSVA’ (29). Differential analysis was conducted using the R package ‘limma’ (30).

### Differentially Expressed Gene Profile and Functional Enrichment Analysis

The ssGSEA score of the hallmark IFNG response of each sample was defined as the IFNG score. Samples were then split into the IFNG score-high and IFNG score-low groups by the median value. Differentially expressed genes (DEGs) (IFNG score-high *vs.* IFNG score-low) of the 3 independent data sets were calculated using the R packages ‘limma’ and ‘edgeR’, respectively (31). The absolute value of log fold change (logFC) > 0.8 and p-value ≤ 0.05 were set as the cut-off. Functional enrichment analysis was performed using the online tool DAVID (https://david.ncifcrf.gov/, v6.8) (32, 33). Gene Ontology (GO) terms including biological process (BP), cellular component (CC), molecular function (MF), and Reactome signaling pathway, were involved in the analysis (34–39).

### Construction and Validation of the IFNGrGS-Based Stratification

The co-upregulated and co-downregulated DEGs were candidate genes for LASSO regression analysis, and regression coefficients were calculated for genes with prognostic significance (40). The IFNGrGS score was calculated for each sample based on the following formula, β_k_ is the regression coefficient of the kth gene.


$$\text{IFNGrGS score}\;=\sum_{k=1}^{n}\beta_{K}\times \text{expression}\;\text{valu}{\text{e}}_{K}$$

The Kaplan-Meier (K-M) analysis was employed to identify differences in overall survival. The time-dependent receiver operating characteristic (ROC) and corresponding area under the curve (AUC) was employed to assess the predictive power of the IFNGrGS-based stratification. The univariate Cox regression analysis was performed to evaluate the independent prognostic value of the IFNGrGS-based stratification.

### Exploring IFNGrGS-Based Stratification-Associated Immunological Characteristics

The immune infiltrates and stromal cells were assessed using the ‘ESTIMATE’ algorithm (41). In particular, the fraction of immune cells of the RNA-seq data sets (TCGA and CGGA325) was evaluated using TIMER, CIBERORT, QUANTISEQ, and XCELL (42–46). The immune infiltration of microarray data (CGGA301) was estimated using CIBERSORT. Based on CIBERSORT, samples with p-values over 0.05 were excluded. To make the correlation between the IFNGrGS score and immune infiltration based on different algorithms comparable, 4 aggregation schemes were defined as follows.

Scheme 1. TIMER
B cell = B cell
CD4 T cell = T cell CD4^+^
CD8 T cell = T cell CD8^+^
Neutrophil = Neutrophil
Macrophage = Macrophage
DC = Myeloid dendritic cell

Scheme 2. CIBERSORT
B cell = B cell naïve + B cell memory + B cell plasma
CD4 T cell = T cell CD4^+^ naïve + T cell CD4^+^ memory resting +T cell CD4^+^ memory activated + T cell regulatory (Tregs)
CD8 T cell = T cell CD8^+^
Neutrophil = Neutrophil
Macrophage = Macrophage M0 + Macrophage M1 + Macrophage M2
Mono/Macro = Monocyte + Macrophage M0 + Macrophage M1 + Macrophage M2
DC = Myeloid dendritic cell resting + Myeloid dendritic cell activated
NK = NK cell resting + NK cell activated
Mast cell = Mast cell resting + Mast cell activated

Scheme 3. QUANTISEQ
B cell = B cell
CD4 T cell = T cell CD4^+^ (non-regulatory) + T cell regulatory (Tregs)
CD8 T cell = T cell CD8^+^
Neutrophil = Neutrophil
Macrophage = Macrophage M1 + Macrophage M2
DC = Myeloid dendritic cell

Scheme 4. XCELL
B cell = B cell + B cell memory + B cell naïve + B cell plasma +
Class-switched memory B cell
CD4 T cell = T cell CD4^+^ memory + T cell CD4^+^ naïve + T cell CD4^+^ (non-regulatory) + T cell CD4^+^ central memory + T cell CD4^+^ effector memory + T cell CD4^+^ Th1 + T cell CD4^+^ Th2 + T cell regulatory (Tregs)
CD8 T cell = T cell CD8^+^ naïve + T cell CD8^+^ + T cell CD8^+^ central memory + T cell CD8^+^ effector memory
Neutrophil = Neutrophil
Macrophage = Macrophage + Macrophage M1 + Macrophage M2
DC = Myeloid dendritic cell activated + Myeloid dendritic cell + Plasmacytoid dendritic cell

The anti-tumor immune response was conceptually divided into 7 stepwise events, including 1.releasing of cancer cell antigens, 2.cancer antigen presentation, 3.priming and activation, 4.trafficking of immune cells to tumors, 5.infiltration of immune cells into tumors, 6.recognition of cancer cells by T cells, and 7.killing of cancer cells. The activity of each step was quantified using the webtool Tumor Immunophenotype Profiling (TIP) (http://biocc.hrbmu.edu.cn/TIP/) (47). Given that T lymphocytes are key players of the anti-tumor immune response, we conducted an extensive literature search to collect chemokines involved in T lymphocyte recruitment (**Supplementary File 2**), as well as immune checkpoints, representing the driving forces that regulate T lymphocytes migration and function, respectively.

### Genomic Alterations Associated With the IFNGrGS-Based Stratification

Somatic mutation data sorted in the form of Mutation Annotation Format (maf) was analyzed using the R package ‘maftools’ (48). The differentially mutated genes (DMGs) were calculated using the function ‘mafCompare’, and mutually exclusive and co-occurring gene pairs were evaluated using the function ‘somaticInteraction’. TMB is defined as the total number of somatic mutations including common substitutions, insertions, and deletions per megabase, and its calculation has been described before (49). The tumor neoantigen burden (TNB) was calculated following Thorsson, V. et al. (26). The tumor purity was estimated by the ABSOLUTE algorithm (50). Significant amplifications and deletions of somatic copy number were detected using GISTIC 2.0 (51).

### Prediction of ICB Responsiveness

Two well-established algorithms, TIDE (http://tide.dfci.harvard.edu/) and ImmuneCellAI (http://bioinfo.life.hust.edu.cn/ImmuCellAI#!/) were employed to predict the clinical response to ICB therapy with default parameters (52, 53). TIDE is a computational framework for modeling the induction of T cell dysfunction in tumors with high infiltration of cytotoxic T lymphocytes and the prevention of T cell infiltration in tumors with low cytotoxic T lymphocyte infiltration level. The TIDE score is correlated with ICB responsiveness. Similarly, the ImmuneCellAI predicts sample responsiveness to ICB by analyzing the expression profiles of ICB responders *versus* non-responders. The SubMap (https://www.genepattern.org/) was employed to validate the reliability of the prediction of TIDE and ImmuneCellAI, which is an unsupervised algorithm revealing common subtypes between independent datasets (54).

### Statistics

Statistical analyses and visualization were performed using the R software v4.0.2 and the online tool ‘hiplot’(https://hiplot.com.cn/). K-M analysis and log-rank test were used to assess survival differences. ROC curves and corresponding AUCs were used to assess the time-dependent predictive power. Univariate Cox regression analysis was used to describe independent prognostic value. Correlation analysis of numerical variables was based on the Pearson or Spearman correlation analysis depending on whether the data is normally distributed. Differences in immune infiltration, ssGSEA score, and TIP score between subgroups were compared using the two-tailed Wilcoxon test. The relationship between the IFNG pathway and its antagonistic pathways and the immune checkpoints were estimated using Pearson correlation analysis and multiple linear regression analysis, ANOVA was employed to test the significance of the model. DMGs and mutually exclusive or co-occurring gene pairs were calculated by Fisher’s exact test. False discovery rate (FDR) was calculated for correction. P-value < 0.05 was considered statistically significant. In the GSEA analysis and the DMG calculation, FDR-q < 0.1 was considered significant. In the functional enrichment analysis, FDR-q < 0.05 was considered significant. We marked * for p < 0.05, ** for p < 0.01, and *** for p < 0.001.

## Results

### IFNG Response Based GBM Clustering

Firstly, the IFNG score was calculated based on the hallmark IFNG response pathway, and samples were then divided into the IFNG score-high and IFNG score-low groups by the median value. DEGs were calculated and GSEA analysis exhibited an enrichment of the hallmark IFNG response pathway in the IFNG score-high group (NES= 1.8, 1.94, and 2.09 in the TCGA, CGGA325, and CGGA301 cohort, respectively) (**Supplementary Figures S1A, B**). Besides, functional enrichment analysis showed that the up-regulated DEGs were enriched in the interferon gamma-mediated signaling pathway (BP) and interferon-gamma signaling (Reactome) (**Supplementary Figure S1C**), indicating that the IFNG score-high group possessed an increased IFNG response.

### The GBM-Based IFNGrGS Was Comparable to Previously Pan-Cancer-Based IFNG Gene Signatures in Characterizing the IFNG Response

**Figure 1:**
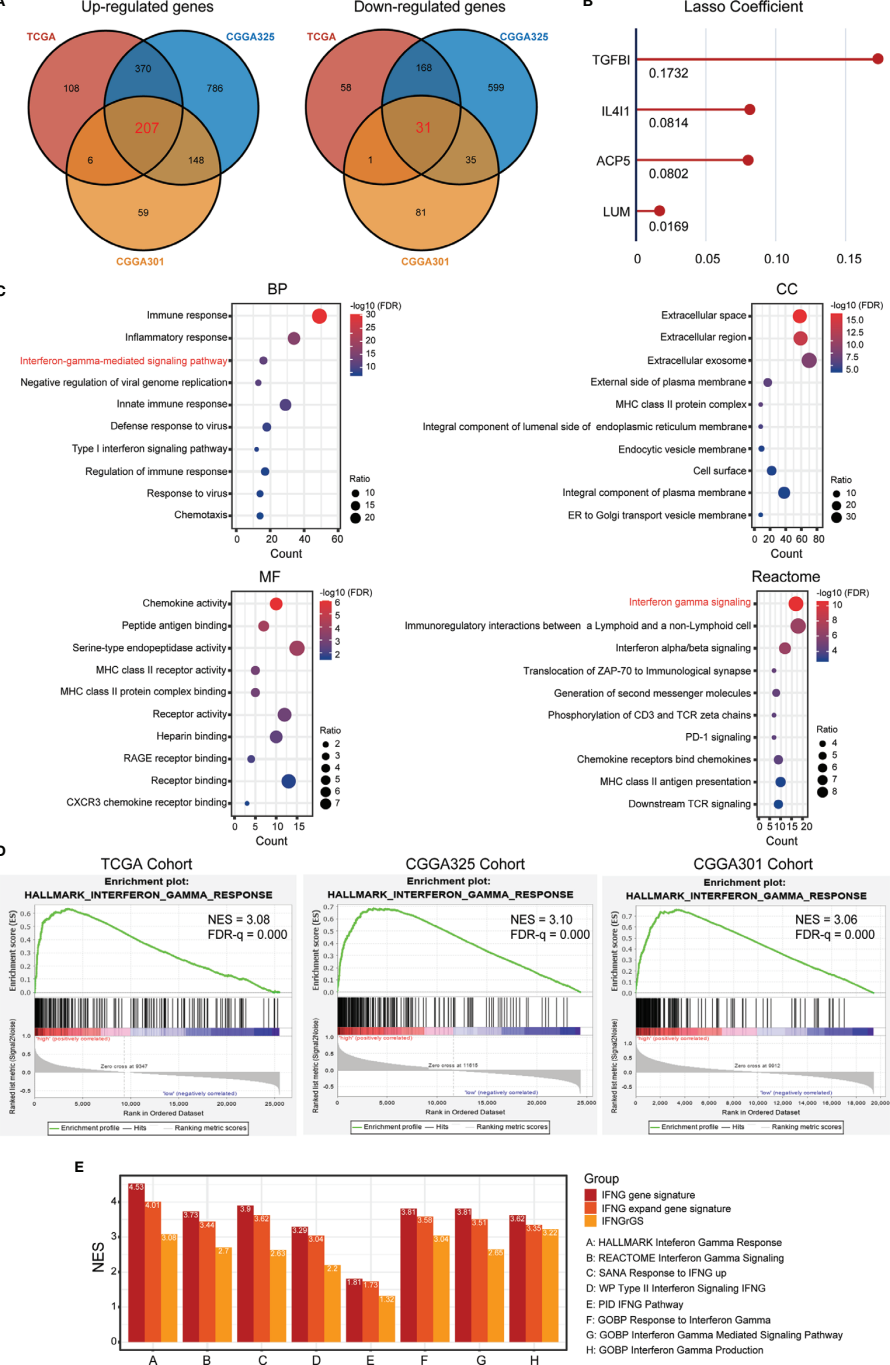
Identification of the IFNG-related gene signature (IFNGrGS) and construction of the IFNGrGS-based stratification. **(A)** Venn diagrams of significantly up-regulated and down-regulated DEGs across 3 data sets. **(B)** The signature genes and corresponding regression coefficients estimated by LASSO. **(C)** Functional enrichment analysis of the 238 DEGs, including BP, CC, MF, and Reactome pathway. **(D)** GSEA analysis of the hallmark interferon-gamma signaling pathway, IFNGrGS score-high group *vs.* IFNGrGGS score-low group. **(E)** Comparison of the efficacy of our IFNGrGS with previously established pan-cancer-based IFNG gene signature and IFNG expand gene signature in characterizing the IFNG response based on the TCGA cohort. NES was calculated using GSEA analysis.

To develop a gene signature effectively characterizing the IFNG response for GBM, DEGs significantly up- and down-regulated across 3 datasets were calculated respectively and intersected to harvest 207 co-upregulated DEGs, as well as 31 co-downregulated DEGs as candidates (**Figure 1A**). Functional enrichment analysis of the 238 genes again revealed enrichment of interferon gamma-mediated signaling pathway (BP) and interferon-gamma signaling (Reactome) (**Figure 1C**). Thereafter, lasso regression analysis identified TGFBI, IL4I1, ACP5, and LUM as significant prognostic factors, which we defined as the IFNGrGS, and determined their regression coefficients for the calculation of the IFNGrGS score (**Figure 1B**). Accordingly, samples were split into the IFNGrGS score-high and -low groups by the median value. GSEA analysis exhibited an increased enrichment of the hallmark interferon-gamma response pathway in the IFNGrGS score-high group (NES = 3.08, 3.10, and 3.06 in the TCGA, CGGA325, and CGGA301 cohort) (**Figure 1D**). Consistently, 9 gene sets associated with IFNG pathway activation were collected and GSVA analysis found that the IFNGrGS score-high group had significantly increased ssGSEA score (**Supplementary Figure S2**), demonstrating higher activation levels. Moreover, a previous study has identified the IFNG gene signature and IFNG expand gene signature for characterizing the IFNG response based on pan-cancer analysis (19). To compare our IFNGrGS with these IFNG gene signatures in characterizing the IFNG response, samples were also split into the IFNG score-high and -low groups (based on the IFNG gene signature) and IFNG expand (IFNGex) score-high and -low groups (based on the IFNG expand gene signature) following the method of Ayers et al. (19). GSEA analysis exhibited comparable NES values of the IFNG pathway in the IFNG score-high, IFNGex score-high, and the IFNGrGS score-high groups, among which the IFNG score-high group being the highest and our IFNGrGS score-high group relatively lower (**Figure 1E**). Together, these results indicated that our constructed IFNGrGS was effective in characterizing the IFNG response in GBM.

### Increased IFNGrGS Scores Were Indicative of Neutrophil and Macrophage Infiltration and Exuberant Innate Immune Response

**Figure 2:**
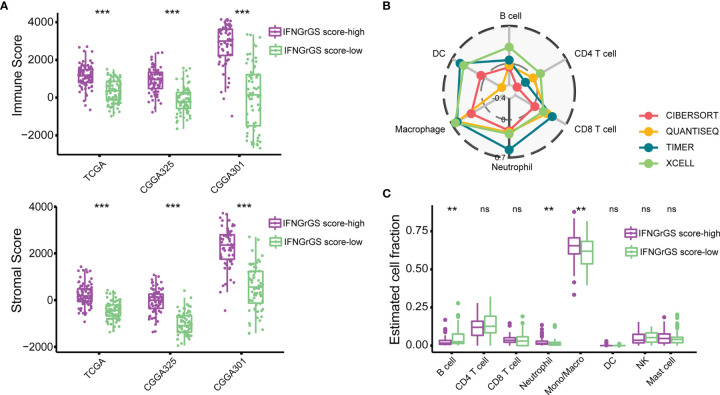
Exploration of the immune infiltration characteristics associated with the IFNGrGS-based stratification. **(A)** The immune-score and stromal-score between the IFNGrGS score-high and -low groups using the ‘ESTIMATE’ algorithm. **(B)** Correlation analysis of the IFNGrGS score and immune infiltrations based on the TCGA cohort. 4 independent *in silico* methods were employed. The correlation coefficient increases from the center (-0.4) to the periphery (0.7), and the grey circle in the middle indicates a correlation coefficient of 0. **(C)** Immune infiltration between the IFNGrGS score-high and -low groups estimated by CIBERSORT based on the TCGA cohort. **p < 0.01, ***p < 0.001, and ns, non-significant.

To explore the IFNGrGS-based stratification-associated immune infiltration pattern, the tumor microenvironment was first evaluated using the ‘ESTIMATE’ algorithm. As a result, the immune score and stromal score of the IFNGrGS score-high group were significantly higher, indicating the aggregation of immune cells and stromal cells in the tumor parenchyma (**Figure 2A**). Further, the fraction of immune infiltration was estimated based on multiple *in silico* methods. We found a robust correlation between neutrophils and macrophages and the IFNGrGS score, with consistently positive correlation coefficients in each algorithm-based group (**Figure 2B** and **Supplementrary Figure S3A**). Notably, the IFNGrGS score-high group had increased neutrophils (in the TCGA and CGGA325 cohort) and monocytes/macrophages (TCGA and CGGA301 cohort), as well as decreased B cells (in the TCGA and CGGA301 cohort) (**Figure 2C** and **Supplementary Figure S3B**), perhaps indicating a relatively hyper-activated innate immune response. This speculation was corroborated by GSVA analysis, where the IFNGrGS score-high group had higher ssGSEA scores on signaling pathways and BPs characterizing inflammation and innate immune response (**Supplementary Figure S4**). However, despite IFNG being one of the most potent macrophage-activating factors (classic activation), we found no significant differences in the infiltration of M1 and M2 macrophages between the IFNGrGS score-high and -low groups (data not shown). Besides, correlation analysis of marker genes of M1/M2 macrophages and microglia with the IFNGrGS score revealed that the genes with higher correlation with the IFNGrGS scores were a mixture of 3 types of genes, further suggesting that M1/M2 type macrophages are co-expressed in the microenvironment of increased IFNG response (**Supplementary Figure S3C**). Overall, these results suggested that an increased IFNGrGS score was associated with an inflammatory tumor microenvironment with increased neutrophil and macrophage infiltration.

### Increased IFNGrGS Score Indicated an Activated but Suppressed Adaptive Immune Response

**Figure 3:**
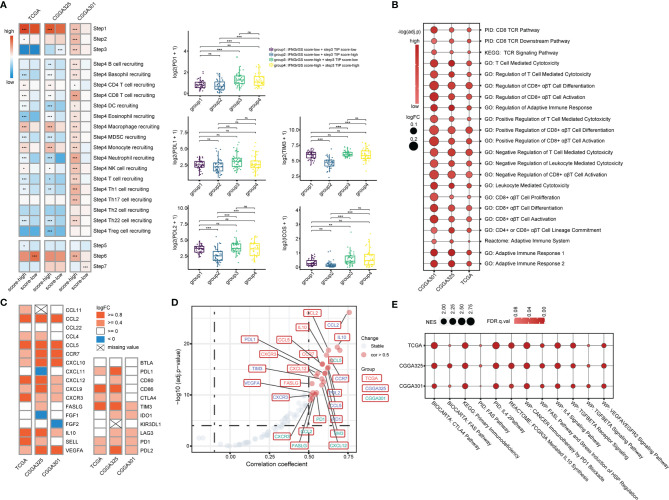
Association between the IFNGrGS-based stratification with the adaptive immune response. **(A)** The TIP system quantifying each step of the anti-tumor immune response. Color represents the average of the TIP score of the IFNGrGS score-high and -low groups. On the right panel, samples were further grouped according to the IFNGrGS score and TIP step3 score to compare the expression of common immune checkpoints (PD1, PDL1, PDL2, TIM3, and ICOS). **(B)** GSVA analysis of pathways involved in the adaptive immune response and CD8 T cell activation. The size of the bubble is proportional to the logFC value (IFNGrGS score-high *vs.* IFNGrGS score-low), color represents the adjust p-value. **(C)** The expression of 17 chemokines regulating the recruitment of the T lymphocyte and 11 co-inhibitory and co-stimulatory molecules regulating the function of T lymphocytes in the tumor niche. Color is proportional to the logFC value. **(D)** The correlation between the IFNGrGS score and the 28 molecules. The absolute value of correlation coefficient > 0.5 and adjust p-value < 0.05 were set as the cut-off. **(E)** The NES values of immunosuppressive pathways including TGF-beta, VEGFA, IL4, IL10, FAS, PD1, CTLA4, and primary immunodeficiency pathway calculated by GSEA analysis. The size of the bubble is proportional to the NES. Color represents the FDR-q value. *p < 0.05, **p < 0.01, ***p < 0.001, and ns, non-significant.

To explore the characteristics of the anti-tumor immune response associated with the IFNGrGS-based stratification, the activity of each step of the anti-tumor immune response was quantified based on the TIP system. As a result, the IFNGrGS score-high group scored significantly higher in tumor antigen release (step1) and immune cell recruitment (step4), but not in T cell priming and activation (step3), immune cell infiltration into tumor (step5), recognition, and destruction of tumor cells (step6, 7) (**Figure 3A**). Notably, samples with low IFNGrGS score and high step3 score (group2) expressed relatively low levels of immune checkpoints, while increased IFNGrGS score was associated with up-regulation of PD1, PDL2, TIM3, and ICOS, indicating that a subset of samples with diminished IFNG response lacks the ability to induce T-cell exhaustion. Besides, ssGSEA analysis of 22 gene sets involved in the activation of CD8 alpha-beta T cell and adaptive immune response found that these pathways scored significantly higher in the IFNGrGS score-high group (logFC > 0 and adjust p-value < 0.001) (**Figure 3B**), with the logFC values of immunosuppressive biological processes were constantly higher than corresponding immuno-stimulatory processes (**Table 1**), possibly revealing an activated but suppressed anti-tumor immune response. We further investigated the expression of chemokines and immune checkpoints that modify the recruitment and function of T lymphocytes and found that CCL2, CCL5, CCR7, CXCL10, and CXCL12, which play a role in recruiting T lymphocytes, and IL10 and VEGFA, that are potential negative regulators of T lymphocyte recruitment, were up-regulated in the IFNGrGS score-high group under the criterion of logFC > 0.8 in at least 2 cohorts (**Figure 3C**). In addition, CD86 (co-stimulatory receptor) and PDL1, TIM3, PD1, and PDL2(co-inhibitory receptor) were also up-regulated in the IFNGrGS score-high group under a relatively lax criterion. Correlation analysis revealed that CCL2, CCL5, CCR7, CXCL12, IL10, VEGFA, FASLG, PD1, and TIM3 were positively correlated with the IFNGrGS score (cor > 0.5 in at least two cohorts) (**Figure 3D**), suggesting sophisticated regulatory paradigms for the adaptive immune response. To this end, GSEA analysis of common signaling pathways that antagonize IFNG found an enrichment of IL4, FAS/FASL, TGF-beta, VEGF, IL10, and immune checkpoint pathways in the IFNGrGS score-high group, with the IL4 pathway (PID), IL4 signaling pathway (WP), cancer immunotherapy by PD1 blockade (WP), CTLA4 pathway (Biocarta), and primary immunodeficiency (KEGG) had average NES values over 2.0 (**Figure 3E**), further validated our conjecture that the adaptive immune response was activated but suppressed with increased IFNGrGS score.

**Table 1:** The logFC values of mutually antagonistic biological processes.

Terms	logFC (high-risk *vs.* low-risk)		
	TCGA	CGGA325	CGGA301
GOBP: positive regulation of CD8 positive alpha beta T cell activation	0.094	0.120	0.285
GOBP: negative regulation of CD8 positive alpha beta T cell activation	0.119	0.187	0.265
GOBP: leukocyte mediated cytotoxicity	0.064	0.090	0.160
GOBP: negative regulation of leukocyte mediated cytotoxicity	0.135	0.107	0.198
GOBP: T cell mediated cytotoxicity	0.075	0.116	0.186
GOBP: negative regulation of T cell mediated cytotoxicity	0.135	0.174	0.198

**Figure 4:**
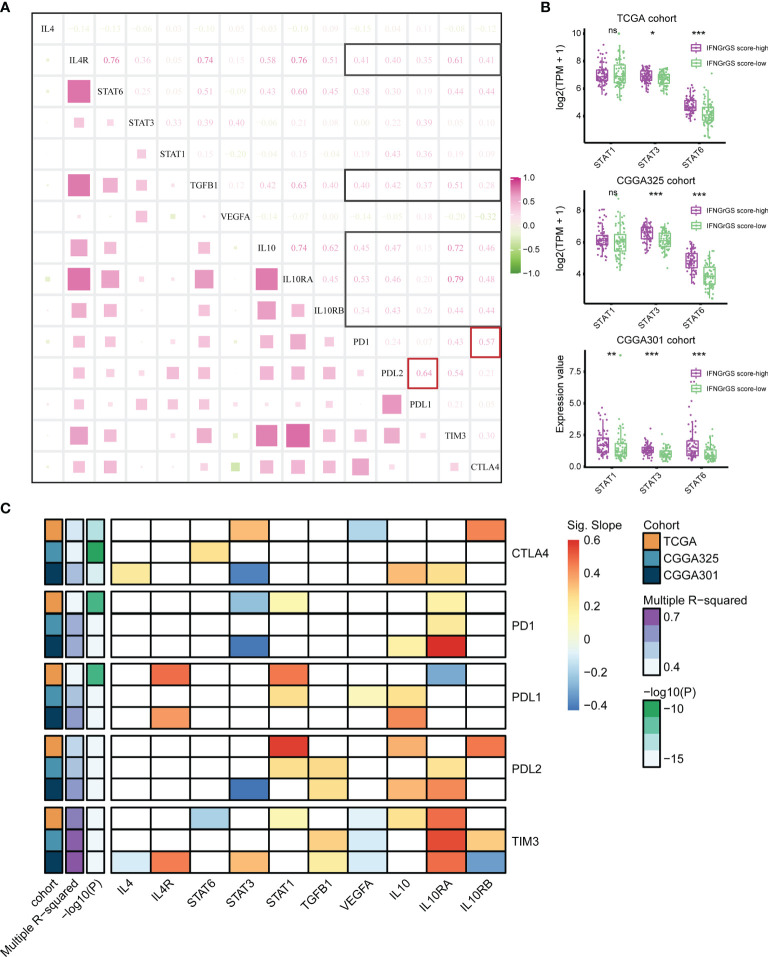
Exploration of the regulatory pattern of immune checkpoint expression in GBM. **(A)** Correlation analysis between the main components of IFNG, IL4, IL10, TGF-beta, and VEGF pathways and immune checkpoints based on the TCGA cohort. Black boxes highlight the correlation of IL4R, TGFB1, and IL10/IL0RA/IL0RB with immune checkpoints. Red boxes highlight the correlation between PD1 and CTLA4, PDL1, and PDL2. **(B)** The expression of STAT1, STAT3, and STAT6 between the IFNGrGS score-high and -low groups. **(C)** Multiple linear regression analysis. The horizontal axis is the putative independent variable that regulates the expression of immune checkpoints and the vertical axis is the dependent variable. The color represents the slope of the calculated linear regression equation which indicates the direction and degree of influence of the independent variable on the dependent variable. Red means that the expression of the dependent variable increases with the independent variable, while blue is the opposite. The white boxes indicate that the effect of the independent variable on the dependent variable is insignificant. Multiple R-squared indicates the extent to which the independent variables determine the expression of the dependent variable. *p < 0.05, **p < 0.01, ***p < 0.001, and ns, non-significant.

Although the CNS was compatible with multiple immunosuppressive mechanisms, their interactions have rarely been dissected. We found significant and extensive correlations between IL4, TGF-beta, IL10, and the immune checkpoints including PD1, PDL1, PDL2, CTLA4, and TIM3 (**Figure 4A** and **Supplementary Figure S5**). Besides, the expression of STAT3 and STAT6, two key transcription factors in the IL4 and IL10 signaling pathways, was significantly higher in the IFNGrGS score-high group (**Figure 4B**), corroborating the activation of IL4 and IL10 signaling pathways. Given that the IFNG-STAT1 signaling pathway is involved in PDL1 expression (55, 56), we presume that the IFNG pathway and pathways counteracting IFNG were all involved in immune checkpoints expression and, therefore, included IL4 (a missing value in the CGGA325 cohort), IL4R, STAT1/3/6, TGFB1, VEGFA, IL10/RA/RB as dependent variables of a multiple linear regression model to interpret the expression of immune checkpoints including PD1, PDL1, PDL2, TIM3, and CTLA4 (**Figure 4C** and **Supplementary File 3**). As a result, the variance inflation factors of all models were less than 5, indicating that the multicollinearity of the models was acceptable. Setting significant results in 2 independent cohorts with coherent direction as the criterion, 3 datasets showed discrete regulatory patterns of CTLA4 expression. IL10RA/STAT3 pathway (TCGA and CGGA301 cohort) may be involved in the expression of PD1. The expression of PDL1 may be co-regulated by STAT1 (TCGA and CGGA325), IL4R (TCGA and CGGA301), and IL10 (CGGA325 and CGGA301), and the expression of PDL2 was co-regulated by STAT1 (TCGA and CGGA325), TGFB1 (CGGA325 and CGGA301), IL10 (TCGA and CGGA301), and IL10RA (CGGA325 and CGGA301), which partially explained the prominent correlation between PDL1 and PDL2 (cor = 0.64, 0.75, and 0.68 in the TCGA, CGGA325, and CGGA301 cohort). Notably, the model explained approximately 70% of TIM3 expression (Multiple R-squared > 0.7), with the most significant effect of IL10 and VEGFA (p < 0.05 across 3 datasets), perhaps indicating that the expression of TIM3 was mainly regulated by IL10 and VEGF signaling pathways. For other immune checkpoints, however, the model R-squareds were always less than 0.6, suggesting the existence of unrevealed mechanisms regulating their expression.

### Genomic Alterations Associated With the IFNGrGS-Based Stratification

**Figure 5:**
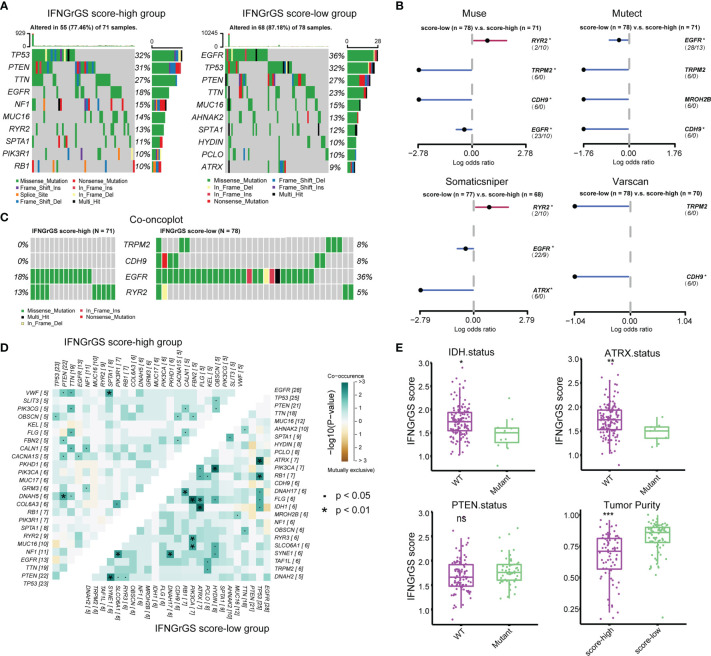
Tumor genomic alterations associated with IFNGrGS-based stratification. **(A)** The top 10 mutated genes in the IFNGrGS score-high and -low groups based on the somatic mutation file ‘mutect’. **(B)** DMGs in the IFNGrGS score-high and -low groups based on 4 somatic callers using Fisher’s exact test. **(C)** The mutation type and frequency of TRPM2, CDH9, EGFR, and RYR2 based on the ‘mutect’ file. **(D)** Co-occurring and mutually exclusive gene pairs. Upper panel: the IFNGrGS score-high group, and lower panel: the IFNGrGS score-low group. **(E)** The association between the IFNGrGS score and IDH, ATRX, and PTEN gene mutation, and the tumor purity. *p < 0.05, **p < 0.01, ***p < 0.001, and ns, non-significant.

Given the profound impact of tumor genomic alterations in the immune-tumor interaction, alterations in single nucleotide polymorphisms (SNP) and copy number variation (CNV) associated with the IFNGrGS score-based stratification were explored. Genes with top mutation frequencies and their predominant mutation types in the IFNGrGS score-high and -low groups were exhibited (**Figure 5A**). We noted gene mutations that are associated with the immune microenvironment and immunotherapy, such as the mutation frequency of PTEN (12), which was 31% in the IFNGrGS score-high group and decreased to 27% in the IFNGrGS score-low group, and the mutation frequency of EGFR (57), which was 18% in the IFNGrGS score-high group and doubled in the IFNGrGS score-low group. TRPM2, CDH9, EGFR (adj-p = 0.089, 0.090, and 0.081 in muse, mutect and somaticsniper), and RYR2 (adj-p = 0.083 and 0.078 in muse and somaticsniper) were DMGs identified by at least two independent somatic callers (**Figure 5B**). The mutation frequency of RYR2 was significantly higher in the IFNGrGS score-high group and the opposite was true for EGFR (**Figure 5C**). Notably, EGFR mutation associated with necrosis and inflammation may be a prognostic risk factor in the IFNGrGS-score low group (p = 0.1) and a protective factor in the IFNGrGS score-high group (p = 0.053) (**Supplementary Figures S6A–C**), suggesting that EGFR gene mutation has heterogeneous prognostic significance for different levels of activation of the IFNG response. Besides, TRPM2/PCLO was a co-occurring gene pair in the IFNGrGS score-low group, indicating a possible synergistic perturbation of different cancer-related biological processes (**Figure 5D**). Moreover, the IFNGrGS score was increased in the IDH and ATRX wildtype samples (**Figure 5E**), corroborating the poor prognosis in the IFNGrGS score-high group. The increased infiltration of immune and stromal cells leads to decreased tumor purity, while CNV, TMB, and TNB did not differ significantly (**Figures 5E** and **Supplementary Figures S6E–G**), suggesting other routes of immune activation in the IFNGrGS score-high group.

### The IFNGrGS-Based Stratification Had a Reliable Prognostic Value

**Figure 6:**
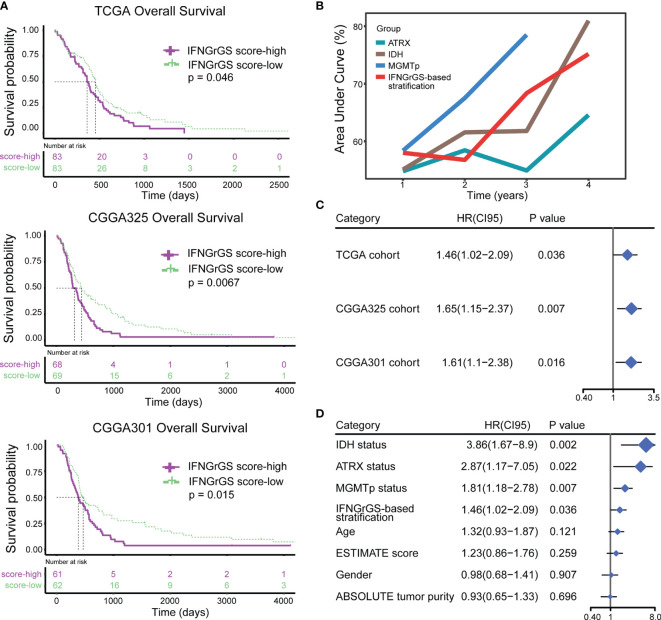
Exploration and validation of the prognostic value of IFNGrGS-based stratification. **(A)** The K-M plots showing the differences in overall survival between the IFNGrGS score-high and -low groups. **(B)** Time-dependent ROC analysis and corresponding AUC values of IDH status (wildtype *vs.* mutant), ATRX status (wildtype *vs.* mutant), MGMT promoter methylation status (unmethylated *vs.* methylated), and IFNGrGS-based stratification (IFNGrGS score-high *vs.* IFNGrGS score-low). **(C)** The independent prognostic value of the IFNGrGS-based stratification (IFNGrGS score-high *vs.* IFNGrGS score-low) based on the univariate Cox regression analysis. **(D)** Comparison of the independent prognostic value of IFNGrGS-based stratification with other molecular pathology parameters based on the TCGA cohort. IDH status: wildtype *vs.* mutant; ATRX status: wildtype *vs.* mutant; MGMTp status: unmethylated *vs.* methylated; Age: > 60 *vs.* <= 60; Gender: male *vs.* female; ESTIMATE score and ABSOLUTE tumor purity: high *vs* low, with the median value being the cut-off.

The prognostic value of the IFNGrGS-based stratification was tested. The K-M analysis showed that the overall survival was significantly reduced in the IFNGrGS score-high group (p-values = 0.046, 0.0067, and 0.015 in the TCGA, CGGA325, and CGGA301 cohort, as well as in other two cohorts) (**Figures 6A** and **Supplementary Figure S7A**). The time-dependent predictive power of the IFNGrGS score-based stratification was also evaluated. In the TCGA cohort, the AUC values of the IFNGrGS score-based stratification of 1-, 2-, 3-, and 4-year survival were 0.58, 0.576, 0.684, and 0.752, which was comparable to the well-studied prognostic biomarkers including IDH and ATRX gene mutation, and MGMT promoter methylation (**Figure 6B**). Univariate Cox regression analysis showed that an increased IFNGrGS score was an independent risk factor (HR = 1.42, 1.65, and 1.61 in the TCGA, CGGA325, and CGGA301 cohort, respectively) with prognostic significance second to IDH and ATRX mutation status, and MGMT promoter methylation status (**Figures 6C, D**). In contrast, the pan-cancer-based IFNG gene signature and IFNG expand gene signature failed to predict prognosis (**Supplementary Figures S7B, C**). These results suggested that the IFNGrGS score-based stratification can be used as a reliable prognostic predictor for GBM.

### IFNGrGS Score-Based Stratification Predicts the ICB Responsiveness and Radiotherapy Efficacy

**Figure 7:**
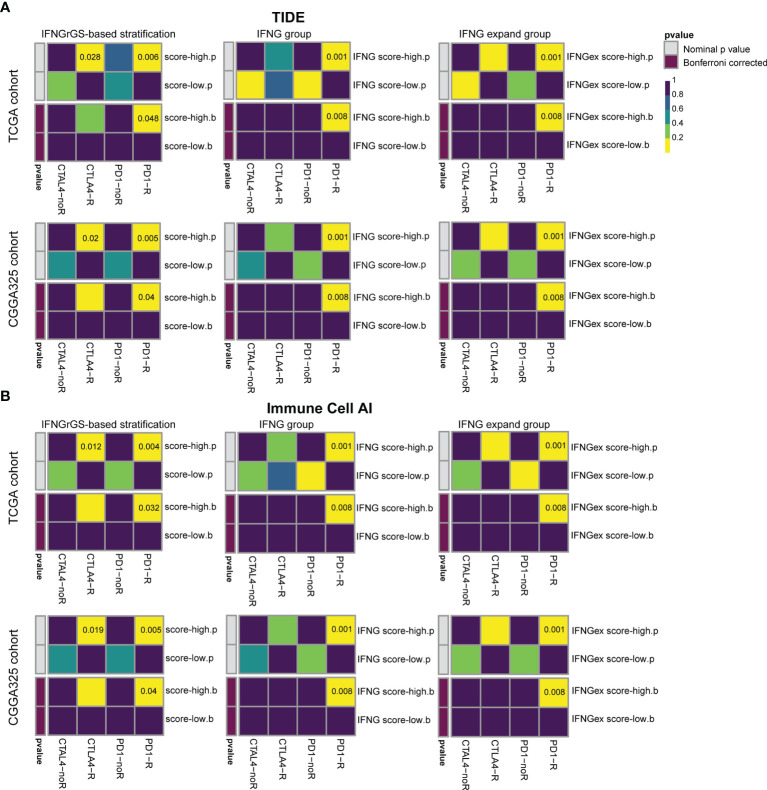
Prediction of the association of IFNGrGS, IFNG gene signature, and IFNG expand gene signature-based stratification with ICB responsiveness. Comparison of the effectiveness of IFNGrGS, IFNG gene signature, and IFNG expand gene signature-based stratification in predicting ICB responsiveness. **(A)** TIDE and **(B)** ImmuneCellAI.

Previous studies have highlighted the role of IFNG gene signatures in predicting the tumor responsiveness to the ICB therapy (19, 20, 58). Given that the IFNGrGS characterized an immune-activated phenotype of GBM, we employed two algorithms (TIDE and ImmuneCellAI) to assess the efficacy of the IFNG-associated gene signatures in predicting ICB responsiveness in GBM and compared the predicted results using SubMap (52–54). As a result, our IFNGrGS had comparable performance to the IFNG gene signature and IFNG expand gene signature in predicting the GBM response to anti-PD1 and anti-CTLA4 therapies (Bonferroni corrected p < 0.05) (**Figure 7**), suggesting that the IFNGrGS based stratification has the potential to identify ICB responders.

**Figure 8:**
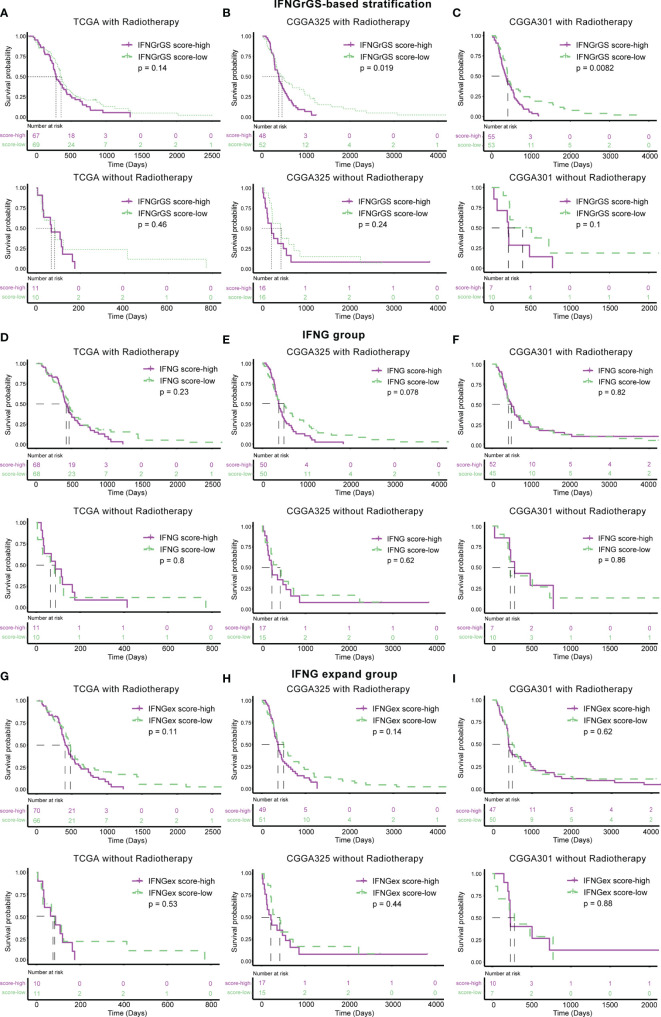
Association of IFNGrGS, IFNG gene signature, and IFNG extended gene signature-based stratification with radiotherapy effectiveness. The association between the IFNGrGS **(A–C)**, IFNG gene signature **(D–F)**, and IFNG expand gene signature **(G–I)**-based stratification and radiotherapy efficacy across 3 data sets.

Further, the association of the 3 IFNG-associated gene signatures with the efficacy of radiotherapy was evaluated. Samples were divided into 2 subgroups according to their radiotherapy status. In the TCGA cohort, the IFNGrGS-based stratification failed to predict the survival benefit for both the patients receiving radiotherapy and those who did not (**Figure 8A**). In the CGGA325 cohort, patients in the IFNGrGS score-low group had significantly prolonged overall survival after receiving radiotherapy (p = 0.019), whereas, for patients who did not, the IFNGrGS-based stratification failed to discern differences in overall survival (**Figure 8B**). Similar results were yielded in the CGGA301 cohort, where a low IFNGrGS score suggested an improved prognosis for patients who received radiotherapy (p = 0.0082) (**Figure 8C**). However, the pan-cancer-based IFNG gene signature and IFNG expand gene signature failed to identify the difference in overall survival between patients who did and did not receive radiotherapy (**Figures 8D–I**). Together, these results suggested that our IFNGrGS performed better than the pan-cancer-based IFNG gene signatures in predicting the efficacy of radiotherapy and that a low IFNGrGS score implied an improved overall survival for GBM patients after radiotherapy.

## Discussion

Effective treatment of GBM remains challenging. Given the paramount role of IFNG in orchestrating the anti-tumor immune response, a comprehensive understanding of the role of IFNG in GBM may help to optimize the current treatment of this disease. Here, we have established a novel IFNGrGS and systemically evaluated the immunological characteristics associated with IFNG response in GBM. In terms of the immune response paradigm, the IFNGrGS score-high group was characterized by significantly increased neutrophil and macrophage infiltration and exuberant innate immune responses, while the activated adaptive immune response was antagonized by multiple immunosuppressive mechanisms. Notably, we have for the first time distilling the molecular mechanisms affecting the expression of multiple immune checkpoints in GBM. In terms of the tumor genome, we identified gene mutations that modifying the tumor immune microenvironment in association with the IFNGrGS-based stratification. Moreover, our IFNGrGS-based stratification had reliable prognostic value and the potential to screen ICB responders and predict radiotherapy efficacy. Overall, this study provides a comprehensive understanding of the intricate impact of IFNG in the special GBM immune microenvironment and a practicable reference for the clinical treatment of GBM.

To our knowledge, we have first established an IFNG-related gene signature in GBM. TGFBI, induced by TGF-beta, encodes an RGD-containing protein that binds to multiple collagens and inhibits cell adhesion (59). Previous studies indicate that TGFBI is associated with glioma proliferation and migration, as well as tumor malignancy (60, 61). Besides, TGF-beta counteracts IFNG by inhibiting CD4 T cell-mediated IFNG production and degrading IFNG mRNA (62, 63). IL4I1 encodes a protein that catabolizes L-phenylalanine and L-arginine. It is expressed by tumor-associated macrophages and plays a role in tumor immune evasion (64). Kynurenic acid mediated by IL4I1 acts as a ligand for AHR, which drives the expression of several molecules mediating the dysfunction of CD8 T cells (65). Interestingly, AHR is involved in the dormancy of tumor cells in response to IFNG, thus promoting tumor immune evasion (66). In addition, AHR induced by IFNG maintains DCs in a tolerant phenotype, impairing antigen presentation (67). ACP5 is an evolutionarily conserved gene and encodes an iron-containing glycoprotein that catalyzes the conversion of orthophosphoric monoester to alcohol and orthophosphate (68). One of the most important positive regulators of ACP5 is IL4, which together with IFNG regulates the balance of Th2/Th1-type responses and avoids damage caused by excessive inflammatory responses (68, 69). LUM encodes a member of a small leucine-rich proteoglycan family that regulates the collagen fibril organization and predicts poor overall survival in gastric cancer (70). Despite the lack of studies revealing a direct relevance between LUM and IFNG response, lumican plays a role in extracellular matrix remodeling, tumor-associated inflammatory response, and GBM resistance to TMZ, and perhaps is involved in the evasion of IFNG killing by GBM (71, 72). Interestingly, these genes were very different from the previously identified IFNG gene signatures that are composed of IFNG signaling and downstream participants, yet were comparative in characterizing the IFNG response in GBM. Therefore, we speculate that these IFNGrGS genes represent the immunosuppressive mechanisms employed by GBM in response to increased IFNG response and thus comprehensively characterize the immune microenvironment with increased IFNG response.

Previous studies have highlighted the anti-GBM effect of IFNG. For example, IFNG combined with retinoid induces apoptosis in T98G and U87MG cells through caspase and calcium-dependent pathways (21). In animal models, exogenous IFNG induces glioma regression, and the anti-tumor effects of IFNG can be reinforced through inhibition of inducible nitric oxide synthase (73, 74). In addition to the direct tumor-killing effects, IFNG, together with IFN-alpha, upregulates the MHC class I molecule expressed on the surface of human GBM cells (75), increasing the immunogenicity of tumor cells. Curiously, little is known about the regulatory role of IFNG on effector T cells in the specific immune context of GBM. We found that the increased IFNG response was accompanied by an active innate immune response, consistent with its pro-inflammatory effects, whereas the adaptive immune response was subject to an intricate regulatory pattern. Classical theory suggests that the immunogenicity of tumors arises from tumor neoantigens originated from accumulated genetic mutations (76). Despite the increased tumor antigen release detected by the TIP scoring system, statistical indicators including SCNA burden, TMB, and TNB were comparable between the IFNGrGS score-high and -low groups, suggesting that the increased tumor antigens in the IFNGrGS score-high group may arise from other sources, such as necrosis. Interestingly, EGFR wild type that was enriched in the IFNGrGS score-high group may be related to the immaturity of the vascular system, tumor tissue hypoxia, and necrosis (57). Given the low immunogenicity of GBM cells and necrosis as a feature of GBM progression (2), the correlation between increased adaptive immune response and poor prognosis may be partially explained. Yet, the relationship between tumor-infiltrating lymphocytes and the prognosis of GBM patients remains controversial (24, 77), leaving an open question of whether anti-tumor immune responses benefit GBM patients.

IL4, IL13, IL10, and TGF-beta are known antagonizers of IFNG. IL4 and IL13 shift the IFNG-mediated inflammation to a wound-healing response in STAT6/STAT3 dependent manner, maintaining the harmony between Th1 and Th2 type immune response (16). In response to IL4/IL13, IFNG not only transiently inhibits these inhibitory pathways but also mediates prolonged refractoriness through epigenetic manners including H3K27 methylation and acetylation (15). IL10 produced by macrophage and Th2 cells inhibits the production of IL12 and IFNG, suppresses Th1-type immune responses, and recruits regulatory T cells (78). Besides, IL10 together with VEGF inhibits T cell migration to the tumor parenchyma by upregulating the expression of Fas ligands in endothelial cells (79). TGF-beta suppresses a variety of processes, including antigen presentation and T cell activation, and impairs NK cell-mediated immune killing by down-regulating the expression of NKG2D ligands (80). Accumulative evidence has suggested the important role of immune checkpoints in disturbing effector T cell function, inducing T lymphocyte dysfunction, and assisting in GBM immune escape (80, 81). Nevertheless, the mechanisms regulating immune checkpoint expression remain obscure. Previous studies have highlighted that IFNG up-regulates PDL1in a STAT1 dependent manner in gastric and colorectal cancer (55, 56). In light of these findings, we investigated the association of the IFNG/STAT1 signaling pathway and signaling pathways that antagonize IFNG with immune checkpoint expression and for the first time revealed that IL4, IL10, TGF-beta and VEGFA may also be involved in the expression of multiple immune checkpoints. The increased IFNG response together with IL4, IL10, VEGF, TGF-beta and immune checkpoints comprise the tumor microenvironment that dominating the immune response, making artificial manipulation of one component only transiently alter the equilibrium reached by such biological response network.

The mutation landscape of glioblastoma has been systemically elucidated. EGFR mutation and vIII mutation are frequent in primary GBM (82). Studies have revealed that EGFR plays a role in regulating the tumor immune microenvironment and immune response. EGFR wild type is associated with decreased expression of BMX/SOX9 and inadequate pericyte coverage in neovascularization, and such an incomplete vascular system exacerbates tumor tissue hypoxia and necrosis. On the other hand, mutations in EGFR leading to elevated PDGFR-beta expression in pericytes can increase infiltration of leukocytes, myeloid cells, and lymphocytes in gliomas (57). Besides, blocking EGFR exacerbates the inflammatory response in the skin, possibly because the presence of EGFR inhibitors enhanced the IFNG-mediated induction of the trans-activator of MHC II (83, 84). These findings are in line with ours that the IFNGrGS score-low group with a higher frequency of EGFR mutations had decreased inflammatory response, implying a relevance between EGFR mutation and IFNG response. PTEN is another GBM driver gene whose mutation is mainly found in the mesenchymal subtype (85). Loss of PTEN results in aberrant tumor cell proliferation depending on c-Met amplification (86). Notably, the enrichment of PTEN mutations and increased PI3K-AKT pathway activity are associated with immunosuppressive mechanisms during ICB treatment of GBM and appear to be enriched in the non-responders (12). Moreover, NF1 acts as a GBM suppressor whose mutation has been reported in a small group of GBM patients (82). NF1 mutation is another characteristic of the mesenchymal subtype and is associated with lymphocytes infiltration (24). Recently, Qianghu Wang et al. have demonstrated that NF1 deletion/mutation drives the recruitment of macrophages, especially M2 (87). Similarly, we have observed enrichment of NF1 mutation and increased macrophage infiltration in the IFNGrGS score-high group. However, the insignificant mutation frequency of NF1 between the IFNGrGS score-high and -low groups suggested that other mechanisms may be involved in the recruitment of macrophages.

The features of the GBM microenvironment characterized by the IFNGrGS contain those of interest, such as IFNG response, activated but suppressed adaptive immune response, and immune checkpoint expression, making the gene signature potentially useful for screening ICB responders. However, other prevalent immunotherapeutic biomarkers like TMB and TNB were not significantly associated with our IFNGrGS-based stratification. Besides, the presence of the blood-brain barrier that is rarely included in the predicting system should greatly diminish the predictive power of our gene signature. Another issue that cannot be ignored in the field of immune-GBM interactions is that the expression of many markers characterizing immune response activity and T-cell infiltration is inversely correlated with the overall survival of glioma patients (20, 58, 88), suggesting that maximizing immune-mediated tumor-killing may not eventually benefit GBM patients. As the adaptive immune response develops from the innate immune response, finding a balance between maximizing overall survival and the immune-mediated tumor clearance may be difficult but interesting if the immune response is both beneficial and detrimental to GBM patients.

In sum, we have constructed a novel clinically valuable IFNGrGS and exhibited a comprehensive view of IFNG-related immunological characteristics of GBM. Although high-throughput sequencing technologies, bioinformatics, and statistical inference are indispensable tools for resolving the intricate interactions between the immune system and tumors and for establishing a theoretical framework, the determination of biological causality and etiology should dependent on cellular and animal experiments, as well as clinical trials. Therefore, subsequent studies are needed to carefully test these findings.

## Data Availability Statement

The original contributions presented in the study are included in the article/**Supplementary Material**. Further inquiries can be directed to the corresponding authors.

## Author Contributions

HJ, JD, and SH conceived and designed the study. HJ and ZL drafted the manuscript. HJ, YB, SMa, TL, QG, JD, and SH revised the manuscript. FW and SL collected the data. HJ, XG, and JJ provided analytical technical support. HJ, KH, and SMi participated in the production of charts and pictures. All authors have read and approved the final manuscript. All authors contributed to the article and approved the submitted version.

## Funding

This work was funded by the National Natural Science Foundation of China (No. 61575058).

## Conflict of Interest

The authors declare that the research was conducted in the absence of any commercial or financial relationships that could be construed as a potential conflict of interest.

## Publisher’s Note

All claims expressed in this article are solely those of the authors and do not necessarily represent those of their affiliated organizations, or those of the publisher, the editors and the reviewers. Any product that may be evaluated in this article, or claim that may be made by its manufacturer, is not guaranteed or endorsed by the publisher.

## Supplementary Material

Supplementary Figure 1 **(A)** DEGs (IFNG score-high *vs.* IFNG score-low) across 3 data sets. **(B)** Enrichment of hallmark IFNG response pathway in the IFNG score-high group. **(C)** Functional enrichment analysis. Top 8 enriched BP terms and Reactome pathway were exhibited. **(D)** The results of lasso regression analysis. *BP, biological process; R, reactome*.

Supplementary Figure 2 Comparison of the ssGSEA scores of IFNG-associated pathways between the IFNGrGS score-high and -low groups. Higher ssGSEA scores indicate increased activity of these signaling pathways in the IFNGrGS score-high group.

Supplementary Figure 3 **(A)** Correlation analysis of the IFNGrGS score and immune infiltration based on the CGGA325 cohort. The correlation coefficient increases from the center (-0.3) to the periphery (0.7), and the grey circle in the middle indicates a correlation coefficient of 0. **(B)** Immune infiltration between the IFNGrGS score-high and -low groups estimated by CIBERSORT based on the CGGA325 and CGGA301 cohorts. **(C)** Scatter plot exhibiting the expression of M1/M2 macrophage and microglia marker genes (logFC was calculated as IFNGrGS score-high/IFNGrGS score-low) and their correlation coefficients with the IFNGrGS score, indicating that increased IFNGrGS scores equivalently recruits M1 and M2 macrophages.

Supplementary Figure 4 The activation of pathways associated with inflammation and innate immune response between the IFNGrGS score-high and -low groups. The increased ssGSEA score indicating that these signaling pathways and BPs were more activated in the IFNGrGS score-high group.

Supplementary Figure 5 Correlation between the main components of IFNG, IL4, IL10, TGF-beta, and VEGF signaling pathways and immune checkpoints. Similarly, IL4R, TGFB1, IL10/IL10RA/IL10RB were extensively associated with the expression of immune checkpoints.

Supplementary Figure 6 **(A–C)** The EGFR mutation had heterogeneous prognostic significance for the IFNGrGS score-high and -low groups. For the IFNGrGS score-low group, EGFR mutation was more of a prognostic risk factor. As for the IFNGrGS score-high group, EGFR mutation was a prognostic protective factor. **(D)** Significant amplification and deletions in copy number. **(E)** Somatic copy number alteration (SCNA) load between the IFNGrGS score-high and -low groups. **(F)** The TMB and **(G)** TNB between the IFNGrGS score-high and -low groups.

Supplementary Figure 7 **(A)** IFNGrGS-based stratification has prognostic value in CGGA693 (RNA-seq) and Gravendeel (microarray) data sets. The K-M plots of previously established pan-cancer-based **(B)** IFNG gene signature and **(C)** IFNG expand gene signature-based stratification. These results indicated that the pan-cancer-based IFNG gene signatures were insensitive in predicting the prognosis of GBM.

Supplementary File 1 Gene sets associated with IFNG response, inflammation and innate immune responses, the adaptive immune response, previously established IFNG gene signatures, as well as M1/M2 and microglia signature genes.

Supplementary File 2 Chemokines recruiting T lymphocytes to the tumor niche.

Supplementary File 3 Multiple linear regression model for explaining the expression of immune checkpoints.

## Abbreviations

IFNG, interferon-gamma; GBM, glioblastoma; IFNGrGS, interferon gamma-related gene signature; ICB, immune checkpoint blockade; CAR-T, chimeric antigen receptor T-cell; CNS, central nervous system; TMB, tumor mutational burden; GSEA, gene set enrichment analysis; GSVA, gene set variation analysis; DEG, differentially expressed gene; GO, gene ontology; BP, biological process; CC, cellular component; MF, molecular function; K-M, Kaplan-Meier; ROC, receiver operating characteristics; AUC, area under the curve; DC, dendritic cell; NK, natural killer cell; TIP, tumor immunophenotype profiling; DMG, differentially mutated gene; TNB, tumor neoantigen burden; NES, normalized enrichment score; SNP, single nucleotide polymorphisms; CNV, copy number variation; HR, hazard ratio; FDR, false discovery rate.
