# Supplementary figures and images for: Construction of Interferon-Gamma-Related Gene Signature to Characterize the Immune-Inflamed Phenotype of Glioblastoma and Predict Prognosis, Efficacy of Immunotherapy and Radiotherapy
Source: Front Immunol. 2021 Sep 10;12:729359. doi: 10.3389/fimmu.2021.729359 (PMC8461254; doi:10.3389/fimmu.2021.729359)

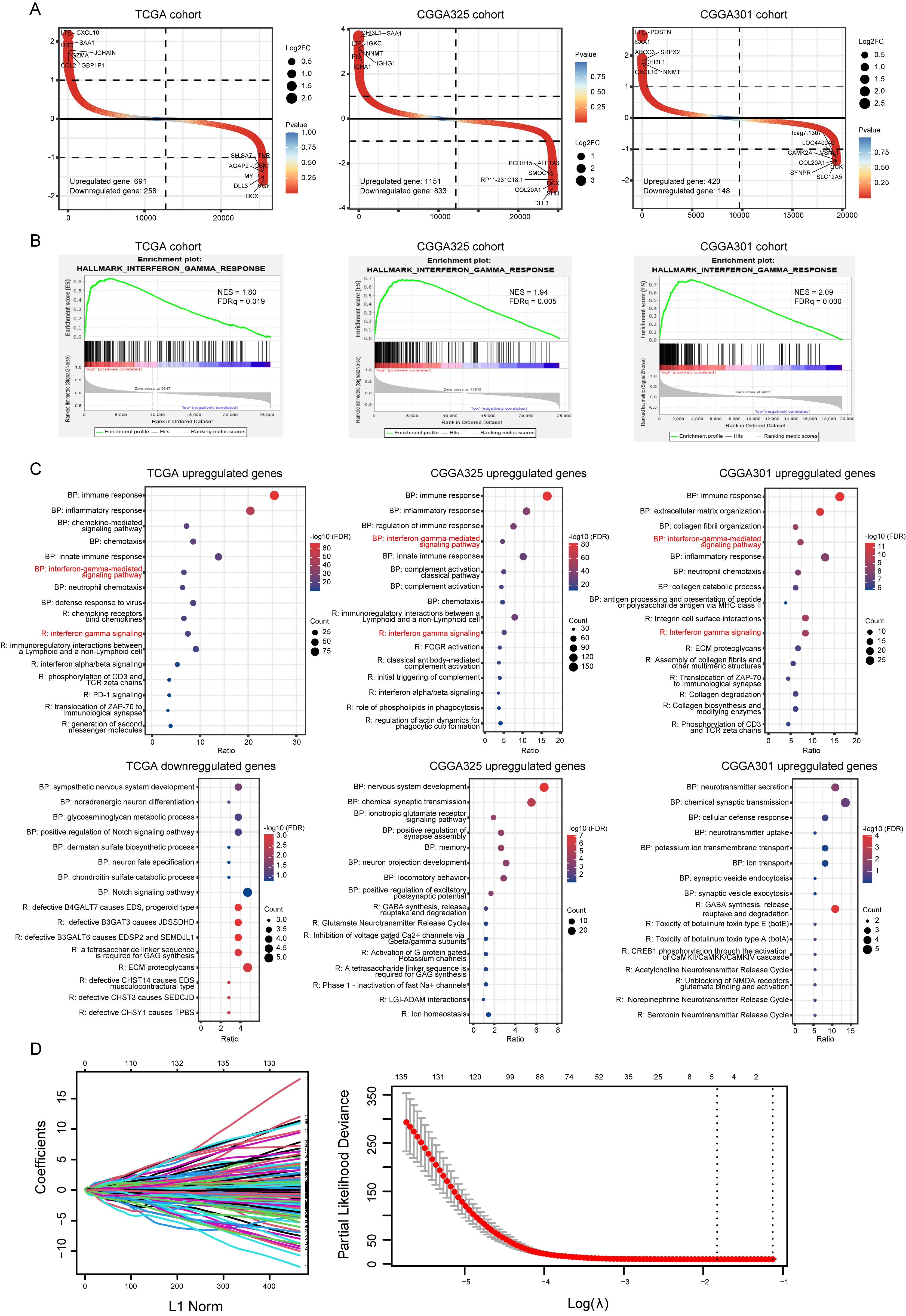

Supplement: Supplementary Figure 1 — (A) DEGs (IFNG score-high vs. IFNG score-low) across 3 data sets. (B) Enrichment of hallmark IFNG response pathway in the IFNG score-high group. (C) Functional enrichment analysis. Top 8 enriched BP terms and Reactome pathway were exhibited. (D) The results of lasso regression analysis. BP, biological process; R, reactome. [file Image_1.tif]

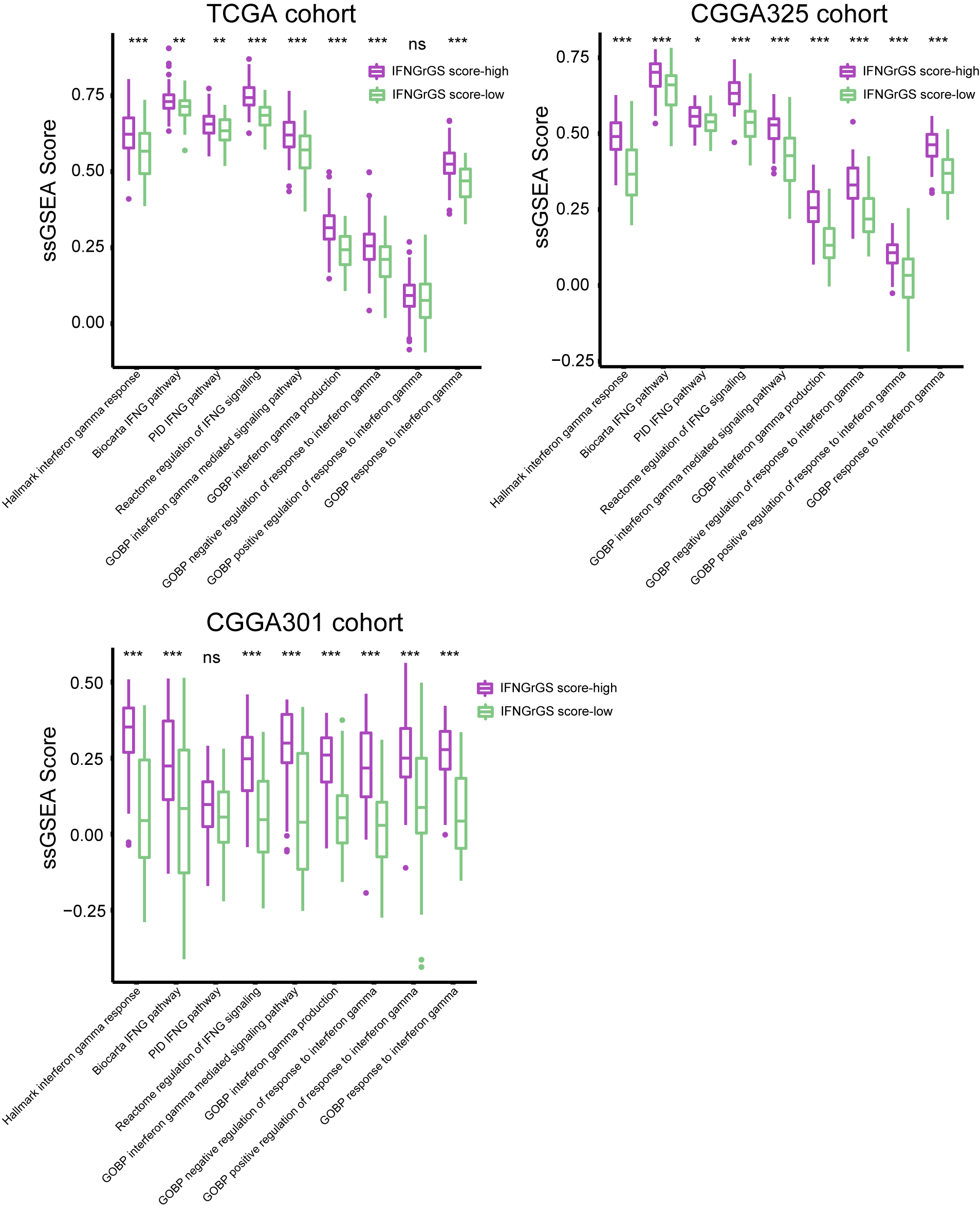

Supplement: Supplementary Figure 2 — Comparison of the ssGSEA scores of IFNG-associated pathways between the IFNGrGS score-high and -low groups. Higher ssGSEA scores indicate increased activity of these signaling pathways in the IFNGrGS score-high group. [file Image_2.tif]

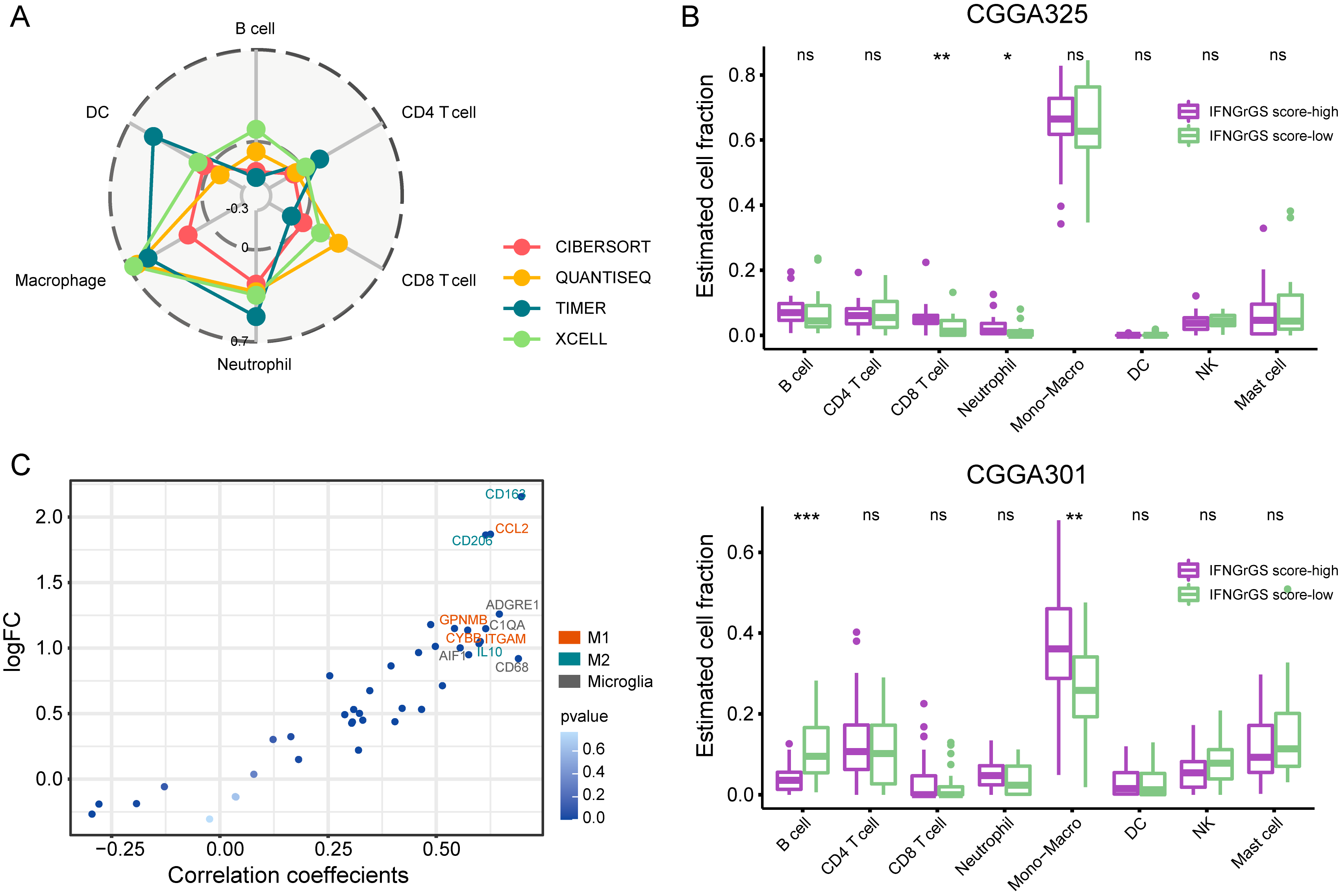

Supplement: Supplementary Figure 3 — (A) Correlation analysis of the IFNGrGS score and immune infiltration based on the CGGA325 cohort. The correlation coefficient increases from the center (-0.3) to the periphery (0.7), and the grey circle in the middle indicates a correlation coefficient of 0. (B) Immune infiltration between the IFNGrGS score-high and -low groups estimated by CIBERSORT based on the CGGA325 and CGGA301 cohorts. (C) Scatter plot exhibiting the expression of M1/M2 macrophage and microglia marker genes (logFC was calculated as IFNGrGS score-high/IFNGrGS score-low) and their correlation coefficients with the IFNGrGS score, indicating that increased IFNGrGS scores equivalently recruits M1 and M2 macrophages. [file Image_3.tif]

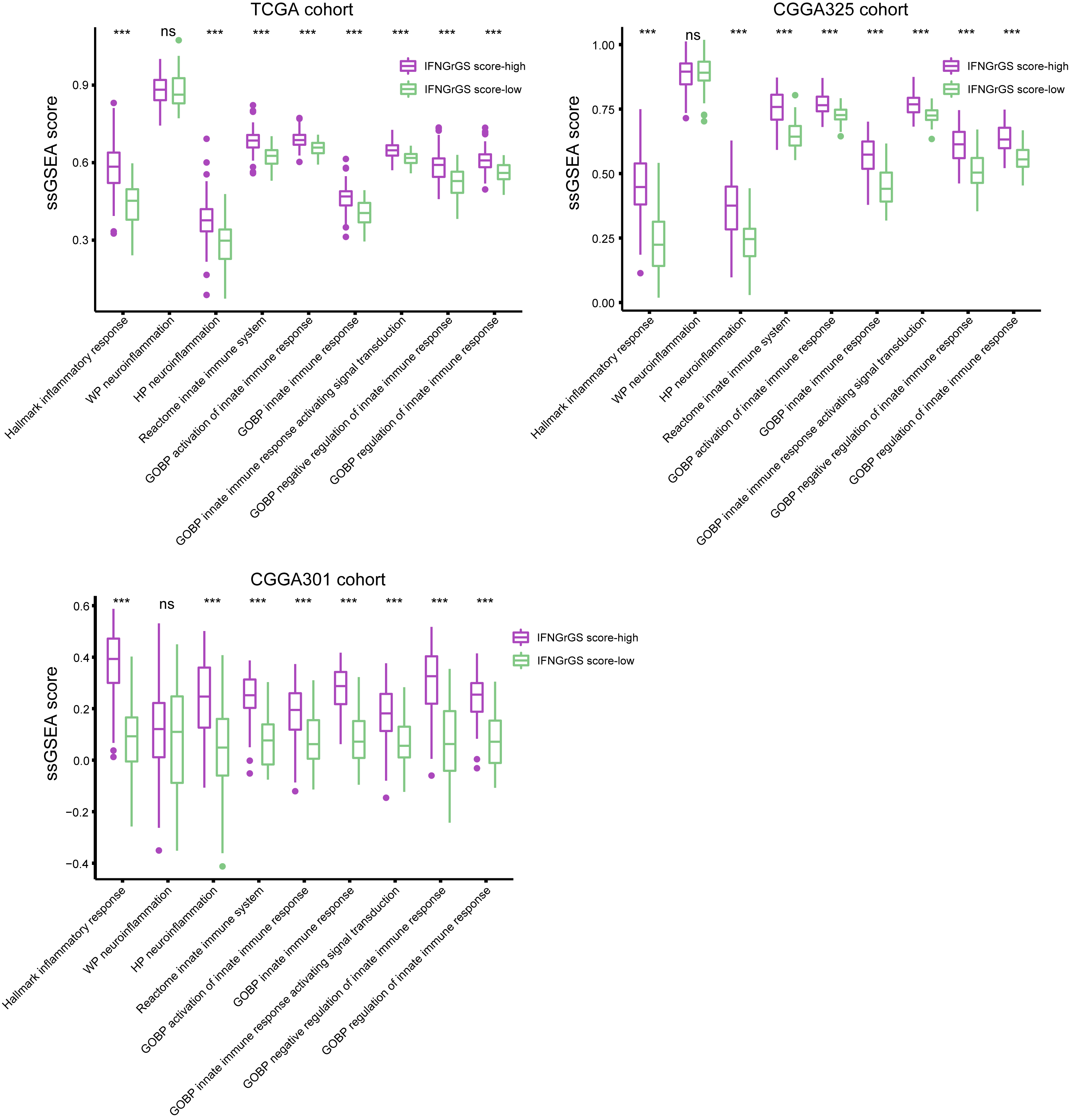

Supplement: Supplementary Figure 4 — The activation of pathways associated with inflammation and innate immune response between the IFNGrGS score-high and -low groups. The increased ssGSEA score indicating that these signaling pathways and BPs were more activated in the IFNGrGS score-high group. [file Image_4.tif]

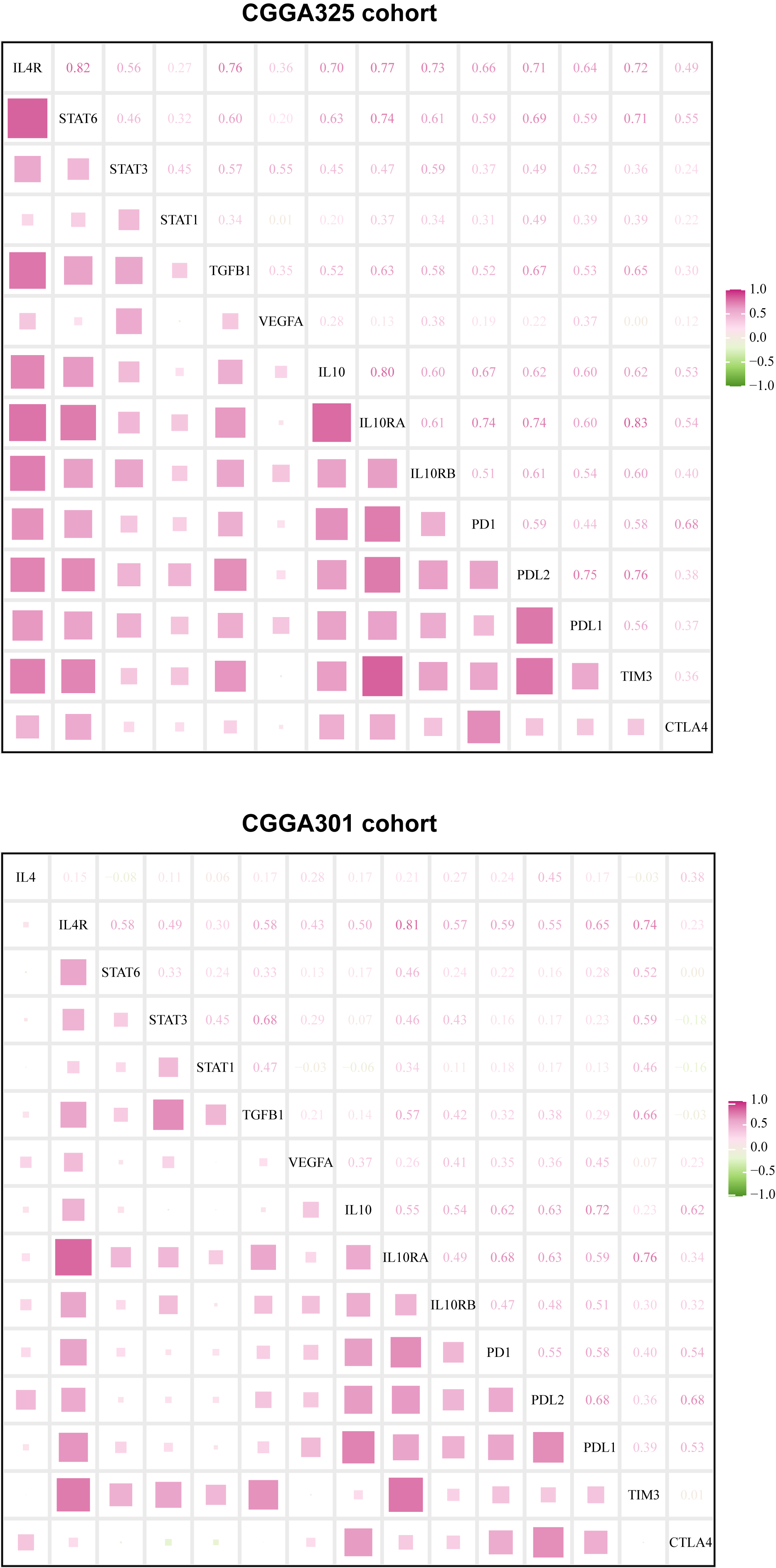

Supplement: Supplementary Figure 5 — Correlation between the main components of IFNG, IL4, IL10, TGF-beta, and VEGF signaling pathways and immune checkpoints. Similarly, IL4R, TGFB1, IL10/IL10RA/IL10RB were extensively associated with the expression of immune checkpoints. [file Image_5.tif]

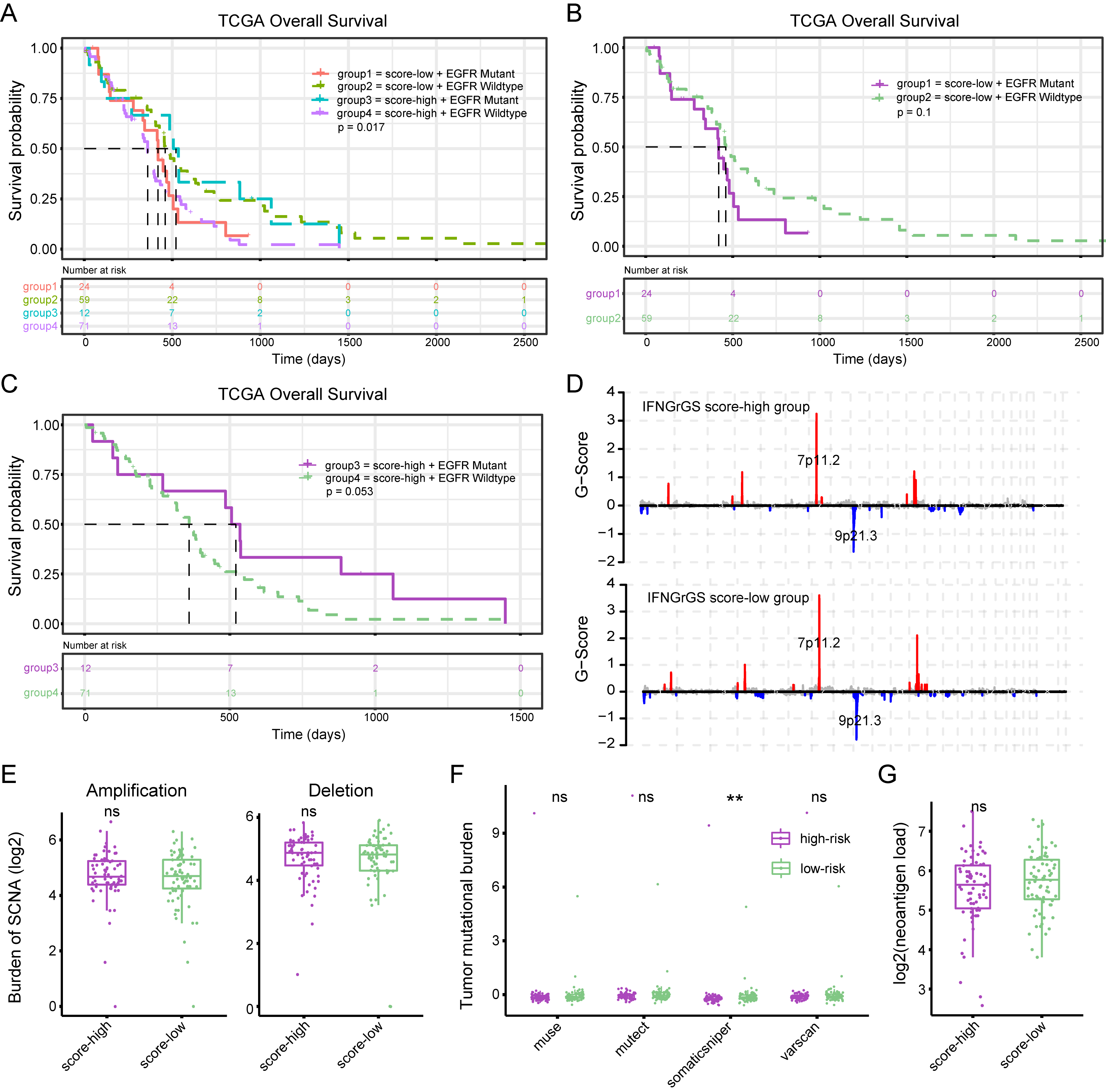

Supplement: Supplementary Figure 6 — (A–C) The EGFR mutation had heterogeneous prognostic significance for the IFNGrGS score-high and -low groups. For the IFNGrGS score-low group, EGFR mutation was more of a prognostic risk factor. As for the IFNGrGS score-high group, EGFR mutation was a prognostic protective factor. (D) Significant amplification and deletions in copy number. (E) Somatic copy number alteration (SCNA) load between the IFNGrGS score-high and -low groups. (F) The TMB and (G) TNB between the IFNGrGS score-high and -low groups. [file Image_6.tif]

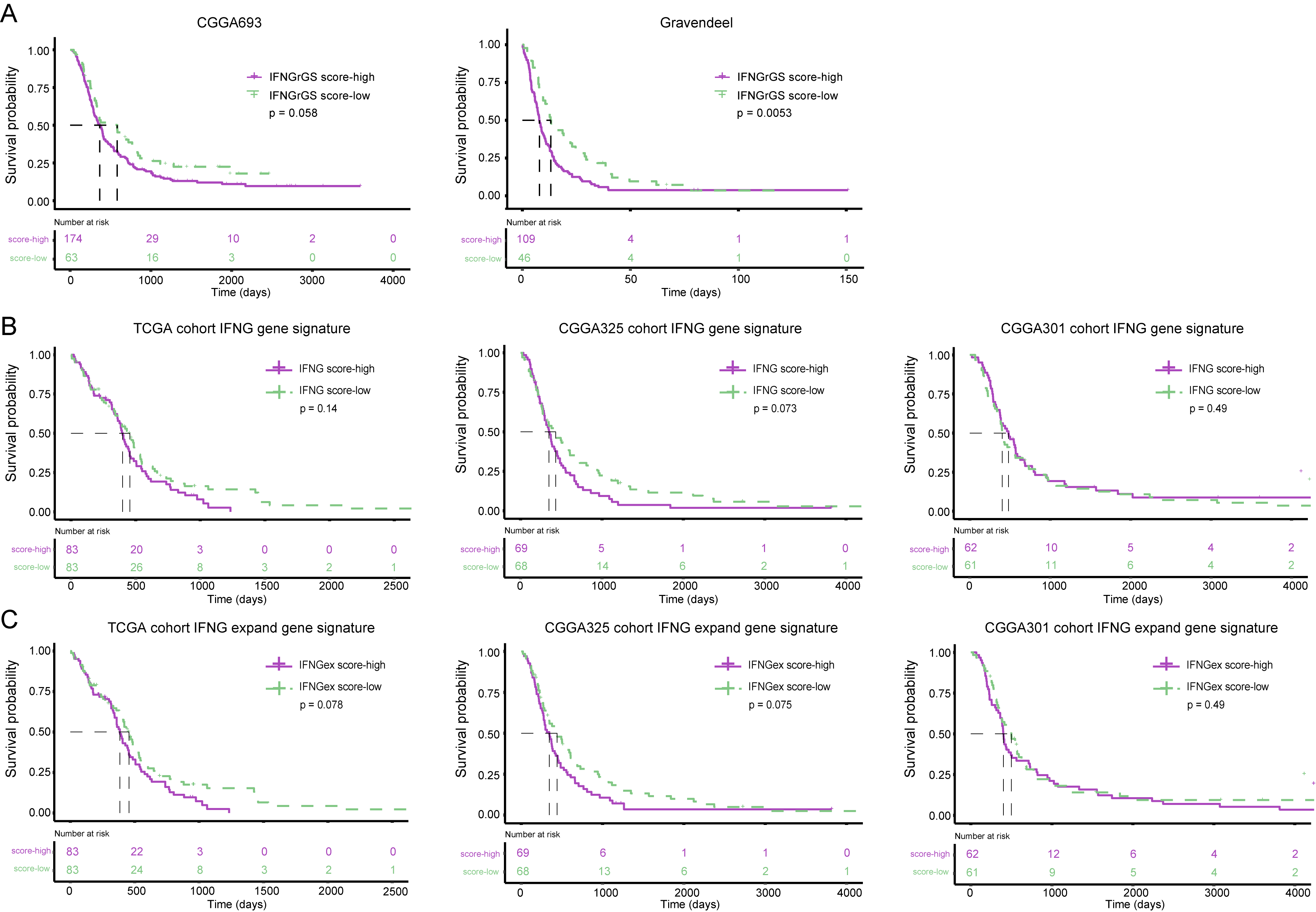

Supplement: Supplementary Figure 7 — (A) IFNGrGS-based stratification has prognostic value in CGGA693 (RNA-seq) and Gravendeel (microarray) data sets. The K-M plots of previously established pan-cancer-based (B) IFNG gene signature and (C) IFNG expand gene signature-based stratification. These results indicated that the pan-cancer-based IFNG gene signatures were insensitive in predicting the prognosis of GBM. [file Image_7.tif]
